# Supplementary material for: Single amino-acid differences define H2B variants and modify chromatin accessibility to induce EMT in breast cancer
Source: Oncogene. 2026 Jan 24;45(6):669–89. doi: 10.1038/s41388-025-03636-1 (PMC12867764; doi:10.1038/s41388-025-03636-1)
Supplement: Supplementary file 2 — Table S1 [file 41388_2025_3636_MOESM2_ESM.pdf]

Dhahri et al. 2025

Single Amino-Acid Differences Define H2B Variants and Modify Chromatin Accessibility to Induce EMT in Breast Cancer

Supplemental Table

Table S1. Key resources table. All relevant materials and resources used in this study.

Table S1

Resource	Source	Identifier or Catalogue Number
**Cell lines**		
MCF10A	ATCC	CRL-10317
MCF7	ATCC	HTB-22
T47D	ATCC	HTB-133
BT474	ATCC	HTB-20
MDA-MB-231	ATCC	HTB-26
MCF10A_EV	This paper	N/A
MCF10A_H2BC_2xHA	This paper	N/A
MCF10A_H2BH_2xHA	This paper	N/A
MCF10A_H2BO_2xHA	This paper	N/A
**Antibodies**		
HA-Tag (C29F4) Rabbit mAb	Cell Signaling	3724
HA-Tag (6E2) Mouse mAb	Cell Signaling	2367
Alexa Fluor 594® anti-Histone H3 Phospho (Ser10) Antibody	BioLegend	650810
Anti-rabbit IgG (H+L), $F(ab')_2$ Fragment (Alexa Fluor® 647 Conjugate)	Cell Signaling	4414
Goat anti-Mouse IgG (H+L) Cross-Absorbed Secondary Antibody, Alexa Fluor™ 680	Invitrogen™	A-21057
Goat anti-Rabbit IgG (H+L) Cross-Adsorbed Secondary Antibody, Alexa Fluor™ 680	Invitrogen™	A-21076
NF-kB p65 (D14E12) XP® Rabbit mAb	Cell Signaling	8242
EGF Receptor (D38B1) XP® Rabbit mAb	Cell Signaling	4267
HDAC2 (3F3) Mouse mAb	Cell Signaling	5113
Histone H3 Antibody	Cell Signaling	9715
Vimentin (D21H3) XP® Rabbit mAb	Cell Signaling	5741
MMP2	Proteintech	10373-2-AP
N-cadherin (D4R1H) XP® Rabbit mAb	Cell Signaling	13116
E-cadherin (24E10) Rabbit mAb	Cell Signaling	3195
GAPDH	Abcam	ab181602
Fibronectin	Cell Signaling	26836
H3K27me3 antibody (C36B11)	Cell Signaling	9733
**Kits**		
MycoSEQ™ Mycoplasma Detection Kits	Applied Biosystems™	4460623

Click-IT™EdU Alexa Fluor™ 488 Flow Cytometry Assay Kit	Invitrogen™	C10425
Subcellular Protein Fractionation Kit for Cultured Cells	Thermo Scientific™	78840
CUTANA™ ChIC/CUT&RUN Kit	EpiCypher	14-1048
iScript cDNA synthesis kit	Bio-Rad	1708891
Pierce™ BCA Protein Assay Kit	Thermo Scientific™	23225
Quick-RNA Miniprep Kit	Zymo Research	R1054
ZymoPURE II Plasmid Maxiprep Kit	Zymo Research	D4202
SF Cell Line 4D-Nucleofector™ X Kit S	Lonza	V4XC-2032
**Bacterial and virus strains**		
Subcloning Efficiency™ DH5α Competent Cells	Invitrogen™	18265017
**Chemicals, Peptides, Recombinant Proteins, and Others**		
DMEM/F12 (no phenol red)	Gibco	21041025
Horse serum	Gibco	16050122
Penicillin-streptomycin (10,000 U/mL)	Gibco	15140122
Hydrocortisone	Sigma Aldrich	H0888
Cholera toxin	Sigma Aldrich	C8052
Insulin	Sigma Aldrich	I-1882
High glucose DMEM (no phenol red)	Gibco	31053028
Fetal Bovine Serum (FBS)	Gibco	A5670701
RPMI 1640 (no phenol red)	Gibco	11835030
MEM (no glutamine, no phenol red)	Gibco	51200038
MEM Amino Acids Solution	Gibco	11130051
MEM Non-Essential Amino Acids Solution	Gibco	11140050
Sodium Pyruvate	Gibco	11360070
Trypsin-EDTA (0.05%)	Gibco	25300054
PBS, pH 7.4	Gibco	10010023
Recombinant Human EGF	PeproTech	315-09-500UG
Recombinant Human TGFβ	PeproTech	100-21-10UG
Recombinant Human TNFα	PeproTech	300-01A-50UG
Rineterkib	MedChemExpress	HY-114491
Puromycin	Sigma-Aldrich	P7255
Paclitaxel	Invitrogen	P3456
Olaparib	MedChemExpress	HY-10162

Doxorubicin	MedChemExpress	HY-15142
4% Formaldehyde	ThermoFisher Scientific Chemicals	J60401.AK
Crystal Violet	Millipore Sigma	C0775
BSA	Millipore Sigma	9048-46-8
Radioisotope α-^{32}P	Revvity	BLU003H250UC
Micrococcal nuclease	NEW ENGLAND Biolabs	M0247S
Proteinase K	NEW ENGLAND Biolabs	P8107S
Exonuclease III	NEW ENGLAND Biolabs	M0206S
DdeI	New England Biolabs	R0175S
DNase I	Zymo Research	E1010
AvaI	NEW ENGLAND Biolabs	R0152S
PstI	NEW ENGLAND Biolabs	R0140S
BamHI	NEW ENGLAND Biolabs	R0136S
Tween 20	Bio-Rad	1662404
Ethanol, Pure (200 Proof, anhydrous)	Millipore Sigma	E7023
Phenylmethylsulphonyl fluoride (PMSF)	Sigma-Aldrich	10837091001
1X HALT protease inhibitor cocktail	Thermo Fisher Scientific	87786
Radioimmunoprecipitation assay (RIPA) lysis and extraction buffer	Thermo Fisher Scientific	J61885.AE
**Oligonucleotides**		
HIST1H2BC 47F	IDT	AGA AGG CAG TGA CCA AAG CGC AG
HIST1H2BC 183R	IDT	GCC CAT GGC CTT GGA AGA GAT GC
HIST1H2BH 87F	IDT	GCG TAA ACG CAG CCG CAA GG
HIST1H2BH 323R	IDT	GCC AGT TCC CCA GGC AGC AG
HIST1H2BO 23F	IDT	TTC ACT CTC CTC CGC CAT GCC C
HIST1H2BO 146R	IDT	CTC TTT GCG GCT GCG CTT GC
TBP F	IDT	TTA TCA ACG CGC GCC AGG G
TBP R	IDT	GGC TGT GGG GTC AGT CCA GT
GAPDH F	IDT	GGA GCG AGA TCC CTC CAA AAT
GAPDH R	IDT	GGC TGT TGT CAT ACT TCT CAT GG
NPS DNA F	IDT	CGGATCCAGAATTCGTGATTGTAGC GTCAACTCACTGCCCTATGCATTATAC AG
NPS DNA R	IDT	GCCGATGCATGTCCCAGTGTCCTCCTT ATGAATCTGAATTCGTCGACAAGC
